# Brain Network Integrity Changes in Subjective Cognitive Decline: A Possible Physiological Biomarker of Dementia

**DOI:** 10.3389/fneur.2021.699014

**Published:** 2021-08-30

**Authors:** Hilla Fogel, Ofri Levy-Lamdan, Noa Zifman, Tal Hiller, Shai Efrati, Gil Suzin, Dallas C. Hack, Iftach Dolev, David Tanne

**Affiliations:** ^1^QuantalX Neuroscience, Beer-Yaacov, Israel; ^2^Sagol Center for Hyperbaric Medicine and Research, Shamir Medical Center, Zerifin, Israel; ^3^Sackler School of Medicine and Sagol School of Neuroscience, Tel-Aviv University, Tel-Aviv, Israel; ^4^Department of Physical Medicine and Rehabilitation, Virginia Commonwealth University, Richmond, VA, United States; ^5^Stroke and Cognition Institute, Rambam Healthcare Campus, Haifa, Israel

**Keywords:** Dementia-Alzheimer's disease, mild cognitive impairment, DELPHI, subjective cognitive decline, plasticity, white matter, gray matter, brain network

## Abstract

**Objective:** The current study seeks to illustrate potential early and objective neurophysiological biomarkers of neurodegenerative cognitive decline by evaluating features of brain network physiological performance and structure utilizing different modalities.

**Methods:** This study included 17 clinically healthy individuals with self-reported cognitive decline (Subjective Cognitive Decline group, SCD, no objective finding of cognitive decline), 12 individuals diagnosed with amnestic Mild Cognitive Impairment (aMCI), 11 individuals diagnosed with Dementia, and 15 healthy subjects. All subjects underwent computerized cognitive performance testing, MRI scans including T1 for gray matter (GM) volume quantification, DTI for quantification of white matter (WM) microstructure fractional anisotropy (FA) and mean diffusivity (MD), and brain network function evaluation using DELPHI (TMS-EEG) measures of connectivity, excitability, and plasticity.

**Results:** Both DELPHI analysis of network function and DTI analysis detected a significant decrease in connectivity, excitability, and WM integrity in the SCD group compared to healthy control (HC) subjects; a significant decrease was also noted for aMCI and Dementia groups compared to HC. In contrast, no significant decrease was observed in GM volume in the SCD group compared to healthy norms, a significant GM volume decrease was observed only in objectively cognitively impaired aMCI subjects and in dementia subjects.

**Conclusions:** This study results suggest that objective direct measures of brain network physiology and WM integrity may provide early-stage biomarkers of neurodegenerative-related changes in subjects that have not yet displayed any other objective measurable cognitive or GM volume deficits which may facilitate early preventive care for neurodegenerative decline and dementia.

## Introduction

Improved medical care and lifestyle continue to increase life span and with it age-related brain disorders (1). The American Academy of Neurology has recently recommended an annual cognitive health assessment for patients 65 years and older (2). The rationale behind this recommendation asserts that brain diseases may be delayed or prevented if detected at early stages and that certain risks associated with compromised brain health are modifiable (3). The lack of effective evaluation tools for brain health leaves most clinical assessments to be based on the patient's clinical history and simple cognitive screening, and the American Academy of Neurology (AAN) has therefore recommended routine assessment of cognitive health in high-risk patients. To date, cognitive assessment has been shown to be the most efficient, clinically usable, and cost-effective tool. Nonetheless, it is well established that objective cognitive manifestations of neurodegeneration are observed only in relatively late stages of disease progression and therefore may not provide the desired preventive effect (4). In contrast, specific physiological changes in brain network connectivity and plasticity are known to occur at the early stages of the disease and therefore it is estimated that a large proportion of cerebrovascular diseases and dementias may be prevented or at least delayed if detected during early pathophysiology stages (5).

In 2014, the term subjective cognitive decline (SCD) was conceived by researchers to describe the subjective experience of worsening cognitive performance among cognitively normal older individuals. SCD has been shown to indicate an *at-risk* stage of Alzheimer's disease (AD) (6). Several studies, using a variety of assessments, found SCD to predict objective cognitive impairment incidents of mild cognitive impairment (MCI) (7, 8) and AD dementia. Furthermore, in several cross-sectional studies, cognitive complaints were found to correlate with biomarkers of AD pathology such as amyloid-ß (Aß) (9). A meta-analysis of longitudinal epidemiological studies of cognitively unimpaired individuals with SCD (with at least 4 years of follow-up data) demonstrated that people with SCD are at increased risk of developing future MCI and that overall the risk of developing dementia is double in individuals having SCD (10). Neuroimaging studies have shown that AD-related changes, including hippocampal volume loss and white matter hyperintensities, already occur in older adults with SCD before objective cognitive impairment becomes evident (11–15), supporting the notion that SCD may provide an indicator of the preclinical phase of AD.

Cognitive complaints may be caused by over 50 known factors such as depression, anxiety, personality factors, and quality of life (16). Improving the characterization of at-risk states and detection of early disease stages are crucial for targeted and effective dementia prevention (1). An ideal method for detecting preclinical, physiological changes reflecting early stages of degeneration would be a patient independent, objective, easily performed, and interpreted method. The current study seeks to illustrate potential early and objective neurophysiological biomarkers of neurodegenerative cognitive decline, by evaluating brain network physiological performance and structure of SCD subjects, aMCI and dementia patients as well as healthy age-related controls, utilizing multiple evaluation paradigms including computerized cognitive performance testing, gray matter (GM) volume, white matter (WM) integrity (Fractional anisotropy and Mean Diffusivity), and brain network function using DELPHI (TMS-EEG) measures of connectivity and plasticity.

## Materials and Methods

### Clinical Data Collection and Analysis

The study was carried out in accordance with the recommendation of the ‘Shamir’ Medical center review board. The protocol was approved by the local institutional “Ethical Committee” as a retrospective study of data. All participants underwent the exact same MRI scan, cognitive battery test, and DELPHI evaluation protocol. The study population included subjects who attended the “Sagol” center for hyperbaric medicine for participating in a study evaluating the effects of hyperbaric oxygen therapy on cognitive performance and health.

### Study Population

The study included 15 healthy, 17 SCD, 12 amnestic MCI (aMCI), and 11 Dementia (most probably Alzheimer's disease) subjects. Inclusion criteria for the Healthy Control (HC) subjects group were as follows: (1) age over 50; (2) no neurological or psychiatric disorder is documented in medical history or self-report; (3) absence of any significant abnormal findings in MRI scan such as brain tumors, subdural hematoma, and other brain-structural lesions related to diagnosed brain disease other than common age-related changes; (4) no psychoactive or other brain-directed medications; (5) no reported Self-experienced persistent decline in cognitive capacity reported during neuropsychologist interview; (6) normal age-, gender-, and education-adjusted performance on standardized cognitive tests. The inclusion criteria for the SCD group are as follows: (1–4) as described in 1–4 for the HC group; (5) subjects that experience a persistent decline in cognitive capacity in comparison with a previously normal status, unrelated to an acute event reported during neuropsychologist interview (16) and were actively seeking medical assistance for their subjective cognitive impairment report; (6) normal age-, gender-, and education-adjusted performance on standardized cognitive tests. The aMCI group was based on Petersen criteria (17), which include memory complaint together with preserved everyday activities, a memory impairment based on a standard neuropsychological test, preserved global cognitive functions, and finally the exclusion of dementia. aMCI inclusion criteria are as follows: (1) age over 50; (2) clinical diagnosis of aMCI by a physician; (3) a Montreal Cognitive Assessment MoCA score 19–25; (4) a computerized testing index score of at least 1.5 standard deviations (STDV) below the age-related norm in verbal and non-verbal memory score only; (5) a normal global standardized cognitive score of Attention/Information Processing/Executive Function; (6) the absence of other unrelated neurological or psychiatric disorders documented in medical history or self-report; (7) the absence of any unrelated significant abnormal findings in MRI scan such as brain tumors, subdural hematoma, and other structural brain lesions. The inclusion criteria for the Dementia group were as follows: (1) age over 50.; (2) a clinical diagnosis of dementia (most probably AD) (18); (3) a Montreal Cognitive Assessment (MoCA) score under 19 as evaluated by neuropsychologist (19, 20); (4) no other major neurological disease; (5) a computerized testing index score of at least 1.5 standard deviations (STDV) below the age-related norm in verbal and non-verbal memory score and at least in one out of three additional computerized tests (Attention/Information Processing/Executive Function); (6) the absence of other unrelated neurological or psychiatric disorder documented in medical history or self-report; (7) the absence of any unrelated significant abnormal findings in MRI scans, such as brain tumors, subdural hematoma, and other structural brain lesions. It is important to note that for all study groups no changes were made to drug treatment regimen during the study duration, subjects were instructed to maintain their routine medical care.

### Computerized Cognitive Evaluation

A computerized cognitive evaluation was performed using NeuroTrax computerized cognitive tests (NeuroTrax Corp., TX) (21). NeuroTrax tests evaluate various aspects of brain functions and include Verbal Memory (immediate and delayed recognition), Non-Verbal Memory (immediate and delayed recognition), Go-No-Go Response Inhibition, Problem Solving, Stroop Interference, Catch Game, and Staged Information Processing Speed (single-digit, two-digit, and three-digit arithmetic). Cognitive index scores were computed from the normalized outcome parameters for memory, executive function (EF), attention, and information processing speed (IPS), (22, 23). After administration, the NeuroTrax data were uploaded to the NeuroTrax central server, and outcome parameters were automatically calculated using software blind to the diagnosis or testing site. To account for the well-known effects of age and education on cognitive performance, each outcome parameter was normalized and fit to an IQ-like scale (mean = 100, S.D. = 15) according to the patient's age and education. The normative data used by NeuroTrax consist of test data from cognitively healthy individuals in controlled research studies at more than 10 sites (24).

### Imaging and Analysis

All subjects underwent MRI scans of the brain and DELPHI (TMS-EEG) evaluations not more than 2 weeks apart. Imaging was performed with a 3 Tesla system (MAGNETOM Skyra, Siemens Healthineers, Erlangen, Germany) with a 20-channel receiver head coil. A weighted T1 analysis was performed with the SPM12 software package (https://www.fil.ion.ucl.ac.uk/spm/), and 30 diffusion-weighted images were scanned with different gradient directions (b = 1,000), one volume without diffusion weighting, using the following parameters: TR = 10,300 ms, TE = 89 ms, Voxel size = 1.8 × 1.8 mm, Matrix = 128 × 128, No. of slices = 63, and Slice thickness = 2.2 mm. Diffuse Tensor Imaging (DTI) analysis was performed on the fractional Anisotrophy (FA) and ADC maps calculated by Siemens post-processing software. For each subject, the WM atlas [ICBM-MORI white matter atlas (25)] was registered to the DTI map using SPM (version 12, The Wellcome Center for Human Neuroimaging, UCL Queen Square Institute of Neurology, London, UK) and manually validated to avoid registration errors. Mean values for FA higher than 0.2 were calculated in WM regions according to the atlas (26–28).

### TMS-EEG

TMS was performed with a MagPro R30 stimulator (MagVenture, Denmark) and an MCF-B65-HO figure-8 Coil (MagVenture, Denmark). The 32-channel EEG data were obtained using TMS compatible BrainAmp DC amplifier (5 kHz sampling rate; ±16.384 mv measurement range; analog low pass filter 1 kHz; Brain Products GmbH, Germany). These were connected to the waveguard™ EEG cap (ANT Neuro, Netherland) with Ag-AgCl electrodes. Electrode impedances were kept below 5 kOhm. The reference and ground electrodes were affixed to the ear lobes. EEG data were recorded using a BrainVision Recorder software (Brain Products GmbH, Germany).

### Experimental Procedure

All TMS stimulations were performed over the left motor cortex (M1). Stimulation protocol, acquisition, and data processing using our fully automated DELPHI algorithm were all performed as described previously by Zifman and Levy-Lamdan et al. (29). TMS coil was positioned over the left cortical motor (M1) region at 45° toward the contralateral forehead according to guidelines (30). Single-pulse (no history dependent; <0.3Hz frequency) and 1 Hz (inhibitory frequency) stimulation were performed at 80% from rest motor threshold (RMT) intensity. Data acquisition, pre-processing, and cleaning of the TMS evoked response include rejection of bad channels and epochs containing large artifacts followed by bandpass FIR filter (0.5–45 Hz) as detailed in Zifman and Levy-Lamdan et al. (29). A thin (0.5 mm) Foam pad was attached to the TMS coil to minimize electrode movement and bone-conducted auditory artifact. Participants were instructed to keep their eyes closed throughout the examination to reduce ocular artifacts. The operator of the system conversed with subjects between the short stimulation protocol blocks in order to avoid drowsiness. Electrodes data were grouped to regional recording hotspots for analysis and statistical purposes: Frontal (F3, F5-ipsilateral and F4, and F6-contralateral to stimulation.); Parietal (C3, C5, CP1-ipsilateral, and C4, C6, and CP2-contralateral to stimulation); Temporal (CP5, CP3, CF5-ipsilateral, and CP6, CP4, and FC6-contralateral to stimulation); Occipital cortex (O1, PO3-ipsilateral, and O2 PO4-contralateral to stimulation).

All data processing, analyses, and statistics were made using MATLAB (R2020a, The Mathworks Inc., MA, United States).

### DELPHI Physiological Network Profile Analysis Parameters

DELPHI analyzed the regional and network TMS-Evoked Potential (TEP) EEG pattern of single and history-dependent events as described in (29). All output measures were calculated as described previously. Waveform Adherence (WFA) refers to single pulse response (20–300 msec after stimulation) adherence to mean healthy age-related signals collected from previous studies. Network Short Term Plasticity is referred to as STP. The normalized difference ratio between the mean field potential of the single pulse response (MFP_single_) and the mean field potential of the inhibitory frequency of stimulation (MFP_i_), indicating network short term plasticity (STP), is calculated as (MFP_single_−MFP_i)_/(MFP_single_+MFP_i_). All data processing and feature extraction were performed automatically by the DELPHI software algorithm.

### Statistical Analysis

Statistical data analysis to account for the differences between groups was made by One Way or Two Way ANOVA with multiple comparison correction by FDR (false discovery rate) test two-stage linear step-up procedure of Benjamini, Kreiger and Yekutieli (Graphpad Prism 9.0) *p < 0.05; **p < 0.01; ***p < 0.001, ns – non-significant.

## Results

**Figure 1:**
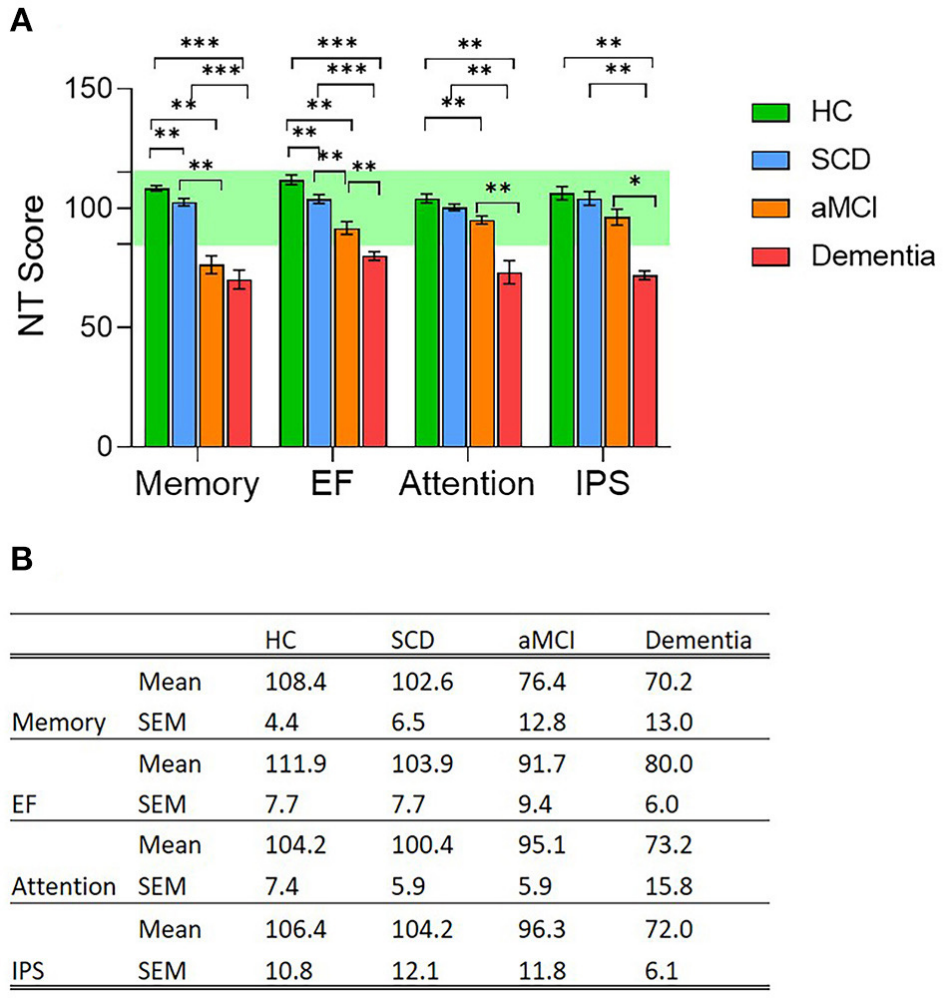
**(A)** Cognitive performance evaluation of four main cognitive domains; memory, Executive Function (EF), attention and information processing speed (IPS); in the four population groups, Healthy Controls (HC, green column); Subjective Cognitive Decline (SCD, blue column); Amnestic Mild Cognitive Impairment (aMCI, orange column), and Dementia (red column). Cognitive evaluation was performed using NeuroTrax computerized cognitive tests (NT). * *p* < 0.05; ** *p* < 0.01; *** *p* < 0.001, ns, non-significant. **(B)** Mean and standard error mean values for each of the four evaluated cognitive domains measured in the four study groups.

The study included 17 clinically healthy individuals with self-reported cognitive decline (Subjective Cognitive Decline group, SCD, no objective finding of cognitive decline), 12 individuals diagnosed with aMCI, 11 individuals diagnosed with Dementia, and 15 healthy subjects; no significant differences were noted between groups ages and the rest motor threshold (RMT) (Table 1). All subjects underwent a thorough evaluation of brain functional performance and health including MRI scans, a computerized cognitive evaluation including four main cognitive domains: memory, executive function (EF), attention, information processing speed (IPS), and DELPHI (TMS-EEG) neurophysiological brain network evaluation. As expected, computerized cognitive evaluation of these cognitive domains did not reveal any abnormal findings in SCD subjects. All SCD subjects scored within healthy population norms. However, compared to the HC group, a small but significant decrease in memory (6 points, *p* < 0.01) and executive function (8 points, *p* < 0.01) was displayed (Figure 1). The aMCI group displayed an abnormal mean memory performance score (decrease of over 1 STD, *p* < 0.01) and a significant (but within the normal range) decrease in EF and attention domains compared to HC (Figure 1; *p* < 0.01). Mean memory and EF performance were significantly lower in the aMCI group compared to the SCD group as well (Figure 1; *p* < 0.01). Dementia subjects displayed abnormal performance in all cognitive domains with a significant decrease compared to the SCD group, and a significant decrease in attention and IPS compared to aMCI subjects (Figure 1; *p* < 0.01; *p* < 0.05 respectively).

**Table 1:** Age, gender, dominant hand, rest motor-threshold (RMT) of the four study groups: Healthy control (HC); Subjective cognitive decline (SCD); Amnestic Mild Cognitive Impaired (aMCI) and Dementia.

	**HC**	**SCD**	**aMCI**	**Dementia**
**N**	15	17	12	11
**Female**	6	4	5	4
**Male**	9	13	7	7
**Left-handed**	none	2	none	none
**Age**	66 ± 6	68.6 ± 8.8	68 ± 6	70.1 ± 4
**Rest Motor-Threshold**	47 ± 8	47.7 ± 10	52 ± 10	50 ± 10

GM volume Region of interest (ROI) analysis of T1 demonstrated no significant decrease in GM volume for SCD compared to healthy norms. The left hippocampus and right Insula GM were significantly lower in aMCI compared to HC (Table 2; *p* < 0.05). Left hippocampus volume was significantly lower while right hippocampus was lower but not significant in aMCI compared to SCD (Table 2; *p* < 0.05, *p* = 0.05 respectively). Other areas did not display any significant changes between SCD and aMCI. GM volume ROI analysis in Dementia subjects displayed a significant decrease in all areas, excluding the left frontal/orbital region, compared to SCD (Table 2; *p* < 0.01), no significant difference was displayed between Dementia and aMCI group (Table 2; *p* < 0.01).

**Table 2:** T1 weighted volume analysis of GM in the four study groups.

		**HC**	**SCD**	**aMCI**	**Dementia**	
**(A) MEAN AND STANDARD ERROR MEAN VALUES FOR EACH OF THE ANALYZED ROIs**						
Frontal inferior\orbital R	Mean	0.65	0.62	0.60	0.57	
	SEM	0.02	0.02	0.03	0.01	
Frontal inferior\orbital L	Mean	0.64	0.60	0.59	0.56	
	SEM	0.02	0.02	0.02	0.01	
Insula right	Mean	0.77	0.74	0.68	0.69	
	SEM	0.02	0.03	0.04	0.02	
Insula left	Mean	0.74	0.70	0.67	0.66	
	SEM	0.02	0.02	0.03	0.02	
Hippocampus right	Mean	0.78	0.79	0.69	0.68	
	SEM	0.02	0.02	0.03	0.03	
Hippocampus left	Mean	0.79	0.80	0.70	0.71	
	SEM	0.02	0.01	0.03	0.01	
Parietal right	Mean	0.57	0.57	0.52	0.53	
	SEM	0.02	0.02	0.03	0.01	
Parietal left	Mean	0.56	0.56	0.52	0.52	
	SEM	0.02	0.01	0.03	0.01	
Temporal right	Mean	0.68	0.68	0.62	0.63	
	SEM	0.02	0.02	0.04	0.01	
Temporal left	Mean	0.68	0.67	0.63	0.62	
	SEM	0.02	0.01	0.03	0.02	
**(B) COMPARISON OF GM VOLUME ANALYSIS BETWEEN EACH PAIR OF STUDY GROUPS**						
**T1**	**HC vs. SCD**	**HC vs. aMCI**	**HC vs. Dementia**	**SCD vs. aMCI**	**SCD vs. Dementia**	**aMCI vs. Dementia**
Frontal inferior\orbital R	0.33	0.19	0.01	0.58	0.04	0.22
Frontal inferior\orbital L	0.26	0.29	0.01	0.87	0.08	0.20
Insula right	0.26	0.03	0.00	0.19	0.02	0.47
Insula left	0.28	0.13	0.01	0.49	0.05	0.32
Hippocampus right	0.91	0.06	0.00	0.05	<0.0001	0.10
Hippocampus left	0.88	0.03	<0.0001	0.03	<0.0001	0.11
parietal right	0.87	0.30	0.01	0.32	0.01	0.19
Parietal left	0.90	0.36	0.01	0.38	0.01	0.15
Temporal right	0.74	0.11	0.01	0.16	0.01	0.42
Temporal left	0.70	0.16	0.02	0.24	0.03	0.44

WM integrity was evaluated by computing FA and MD for major ROIs including Corpus Callosum (CC), Corona Radiata, Cingulum, Fornix, and Fronto-Occipital fibers. DTI-FA analysis demonstrated a significant decrease in WM fibers FA between HC and SCD for the CC, anterior Corona Radiata, Superior Longitudinal Fasciculus, Superior Fronto-Occipital Fasciculus, and right Hippocampus (Table 3; *p* < 0.05). The left Hippocampus displayed lower but non-significant FA measures in SCD compared to HC (Table 3; *p* = 0.05). Significantly lower FA values were measured in the aMCI group compared with HC in most ROI, excluding the superior Corona Radiata. aMCI of the right Hippocampus, left Fornix, and splenium of CC displayed a lower but non-significant difference from HC (Table 3; *p* = 0.07). No significant change was observed between the SCD and aMCI groups and between Dementia subjects group to both SCD and aMCI groups. As expected, Dementia subjects displayed significantly lower FA values for all measured ROIs compared to HC (Table 3; *p* < 0.05), excluding superior Corona Radiata, left anterior Corona Radiata, right Hippocampus, and right Fornix.

**Table 3:** DTI- fractional anisotropy (FA) analysis of WM fibers in the four study groups.

	**HC**		**SCD**		**aMCI**		**Dementia**	
**DTI-FA**	**Mean**	**SEM**	**Mean**	**SEM**	**Mean**	**SEM**	**Mean**	**SEM**
**(A) MEAN AND STANDARD ERROR MEAN VALUES FOR EACH OF THE ANALYZED ROIs**								
Genu of corpus callosum'	0.81	0.01	0.72	0.04	0.71	0.02	0.68	0.02
Body of corpus callosum'	0.87	0.01	0.79	0.04	0.79	0.02	0.75	0.03
Splenium of corpus callosum'	0.95	0.01	0.87	0.04	0.88	0.02	0.81	0.03
Anterior corona radiata L'	0.67	0.01	0.60	0.01	0.58	0.02	0.60	0.02
Anterior corona radiata R'	0.69	0.01	0.62	0.01	0.59	0.03	0.61	0.03
Superior corona radiata L'	0.73	0.01	0.68	0.01	0.68	0.02	0.70	0.03
Superior corona radiata R'	0.73	0.01	0.68	0.01	0.68	0.02	0.68	0.03
Cingulum (hippocampus) L'	0.74	0.01	0.68	0.03	0.66	0.02	0.66	0.02
Cingulum (hippocampus) R'	0.71	0.02	0.65	0.03	0.64	0.02	0.67	0.02
Fornix L'	0.75	0.01	0.70	0.01	0.68	0.02	0.67	0.02
Fornix R'	0.75	0.02	0.69	0.01	0.66	0.02	0.69	0.02
Superior longitudinal fasciculus L'	0.77	0.02	0.69	0.03	0.67	0.02	0.69	0.02
Superior longitudinal fasciculus R'	0.75	0.02	0.68	0.03	0.65	0.02	0.67	0.02
Superior fronto-occipital L'	0.79	0.01	0.67	0.02	0.71	0.03	0.68	0.05
Superior fronto-occipital R'	0.79	0.03	0.68	0.03	0.68	0.04	0.65	0.04
**(B)** ***P*** **-VALUES OF DTI-FA ANALYSIS COMPARISON BETWEEN EACH PAIR OF STUDY GROUPS**								
**DTI-FA** ***p*** **value**	**HC vs. SCD**	**HC vs. aMCI**	**HC vs. Dementia**	**SCD vs. aMCI**	**SCD vs. Dementia**	**aMCI vs. Dementia**		
Genu of corpus callosum'	0.01	0.02	0.00	0.96	0.31	0.42		
Body of corpus callosum'	0.01	0.04	0.00	0.99	0.30	0.39		
Splenium of corpus callosum'	0.01	0.07	0.00	0.87	0.08	0.12		
Anterior corona radiata L'	0.04	0.02	0.06	0.54	0.97	0.60		
Anterior corona radiata R'	0.02	0.02	0.03	0.57	0.84	0.73		
Superior corona radiata L'	0.13	0.24	0.40	0.97	0.62	0.70		
Superior corona radiata R'	0.17	0.23	0.28	0.91	0.91	0.84		
Cingulum (hippocampus) L'	0.05	0.03	0.03	0.53	0.63	0.87		
Cingulum (hippocampus) R'	0.04	0.07	0.26	0.82	0.51	0.45		
Fornix L'	0.13	0.07	0.02	0.54	0.29	0.75		
Fornix R'	0.07	0.03	0.10	0.42	0.96	0.50		
Superior longitudinal fasciculus L'	0.01	0.02	0.03	0.68	0.97	0.69		
Superior longitudinal fasciculus R'	0.02	0.01	0.03	0.54	0.91	0.65		
Superior fronto-occipital L'	0.00	0.04	0.00	0.41	0.98	0.48		
Superior fronto-occipital R'	0.00	0.01	0.00	0.91	0.33	0.48		

Mean Diffusivity (MD) measures of WM integrity support FA measures, demonstrating increased MD in the SCD group compared to HC for Genu of the CC, Fornix, Hippocampus, and Fronto-Occipital Fasciculus. The aMCI group's MD is increased in the splenum of CC and fornix alone compared to HC (Table 4; *p* < 0.05). No significant difference was observed between SCD and aMCI groups. However, the dementia group displayed significantly higher MD in the splenum of CC (*p* < 0.01) and body of CC (*p* = 0.05) compared to SCD (Table 4). Significantly higher MD values were measured in the Dementia group compared with HC in the CC, Fornix, and Fronto-Occipital Fasciculus, (Table 4; *p* < 0.05).

**Table 4:** DTI- Mean Diffusivity (MD) analysis of WM fibers in the four study groups.

	**HC**		**SCD**		**aMCI**		**Dementia**	
**DTI-MD**	**Mean**	**SEM**	**Mean**	**SEM**	**Mean**	**SEM**	**Mean**	**SEM**
**(A) MEAN AND STANDARD ERROR MEAN VALUES FOR EACH OF THE ANALYZED ROIs**								
Genu of corpus callosum'	0.35	0.01	0.40	0.03	0.39	0.01	0.44	0.02
Body of corpus callosum'	0.33	0.01	0.36	0.02	0.37	0.01	0.41	0.03
Splenium of corpus callosum'	0.31	0.01	0.32	0.01	0.36	0.01	0.40	0.03
Anterior corona radiata L'	0.26	0.00	0.30	0.01	0.29	0.01	0.30	0.01
Anterior corona radiata R'	0.26	0.00	0.29	0.01	0.29	0.01	0.31	0.02
Superior corona radiata L'	0.25	0.00	0.27	0.01	0.28	0.01	0.29	0.01
Superior corona radiata R'	0.25	0.00	0.27	0.01	0.28	0.01	0.29	0.01
Cingulum (hippocampus) L'	0.28	0.00	0.33	0.03	0.31	0.00	0.32	0.01
Cingulum (hippocampus) R'	0.28	0.00	0.33	0.03	0.31	0.01	0.32	0.01
Fornix L'	0.32	0.01	0.37	0.01	0.38	0.01	0.39	0.02
Fornix R'	0.33	0.01	0.37	0.01	0.39	0.01	0.39	0.02
Superior longitudinal fasciculus L'	0.25	0.00	0.28	0.01	0.27	0.00	0.28	0.01
Superior longitudinal fasciculus R'	0.25	0.00	0.28	0.01	0.28	0.00	0.28	0.01
Superior fronto-occipital L'	0.26	0.00	0.32	0.02	0.30	0.01	0.33	0.02
Superior fronto-occipital R'	0.27	0.01	0.32	0.01	0.31	0.01	0.34	0.02
**(B)** ***P*** **-VALUES OF DTI-MD ANALYSIS COMPARISON BETWEEN EACH PAIR OF STUDY GROUPS**								
**DTI-MD** ***p*** **Value**	**HC vs. SCD**	**HC vs. aMCI**	**HC vs. Dementia**	**SCD vs. aMCI**	**SCD vs. Dementia**	**aMCI vs. Dementia**		
Genu of corpus callosum'	0.01	0.08	0.00	0.70	0.19	0.14		
Body of corpus callosum'	0.10	0.13	0.00	0.87	0.05	0.13		
Splenium of corpus callosum'	0.42	0.04	0.00	0.16	0.00	0.15		
Anterior corona radiata L'	0.12	0.22	0.11	0.93	0.79	0.76		
Anterior corona radiata R'	0.11	0.21	0.05	0.93	0.52	0.53		
Superior corona radiata L'	0.26	0.32	0.13	0.95	0.55	0.65		
Superior corona radiata R'	0.31	0.30	0.13	0.85	0.51	0.69		
Cingulum (hippocampus) L'	0.02	0.33	0.17	0.30	0.50	0.74		
Cingulum (hippocampus) R'	0.03	0.29	0.21	0.45	0.53	0.89		
Fornix L'	0.02	0.03	0.01	0.79	0.57	0.81		
Fornix R'	0.04	0.02	0.03	0.56	0.63	0.91		
Superior longitudinal fasciculus L'	0.20	0.35	0.26	0.87	1.00	0.89		
Superior longitudinal fasciculus R'	0.20	0.26	0.21	0.96	0.89	0.94		
Superior fronto-occipital L'	0.01	0.12	0.01	0.56	0.56	0.30		
Superior fronto-occipital R'	0.04	0.13	0.01	0.84	0.29	0.28		

**Figure 2:**
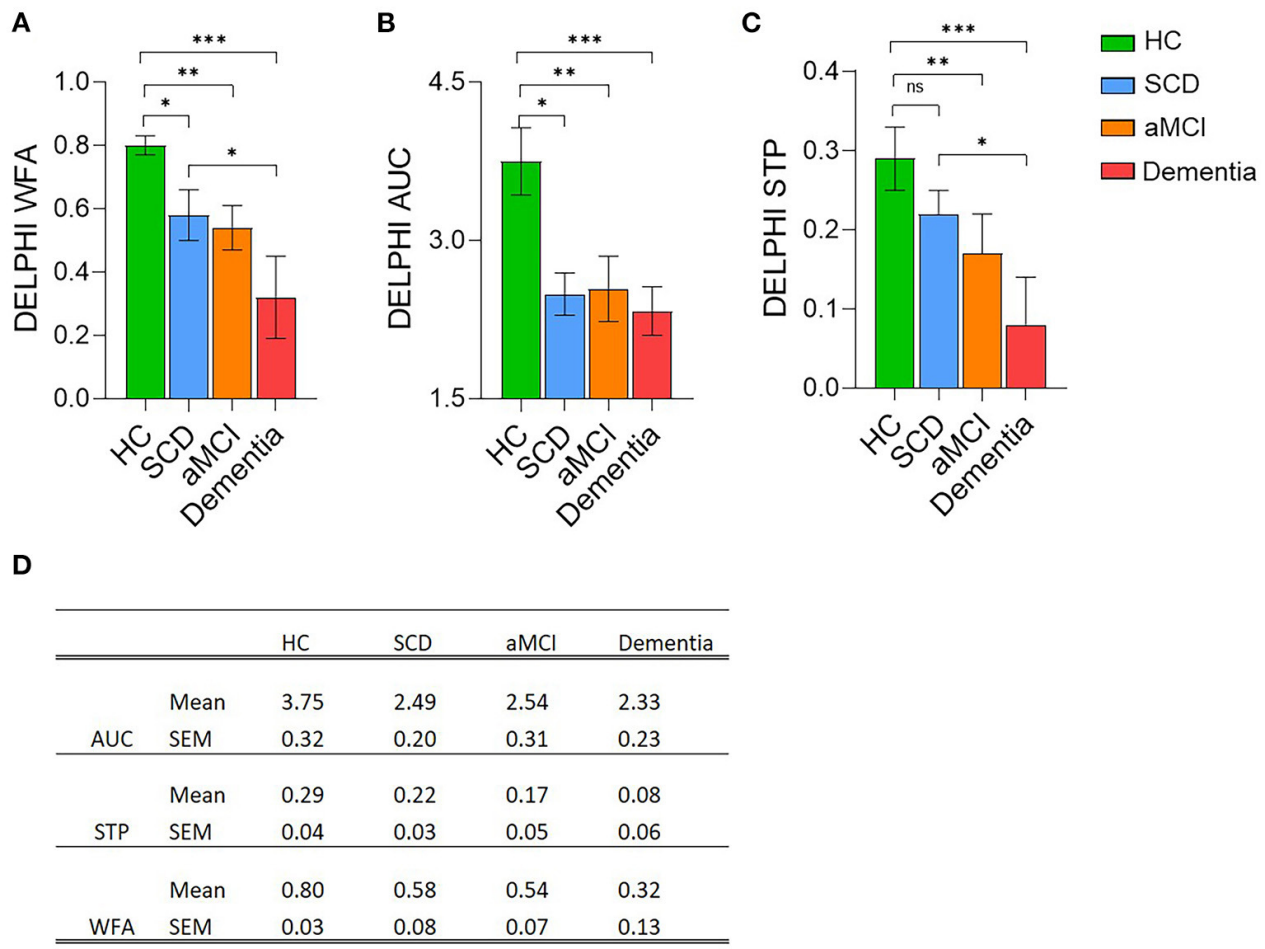
DELPHI analysis of TEP response analysis mean values of the four cognitive decline populations. Healthy Controls (HC, green column); Subjective Cognitive Decline (SCD, blue column); Amnestic Mild Cognitive Impairment (aMCI, orange column), and Dementia (red column). **(A)** DELPHI TEP measure of Waveform Adherence (WFA). **(B)** DELPHI TEP measure of Area Under the Curve (AUC). **(C)** DELPHI TEP measure of Short Term Plasticity (STP). *p < 0.05; **p < 0.01; ***p < 0.001, ns, non-significant. **(D)** Mean and standard error mean values for each of the DELPHI TEP measures in the four study groups.

DELPHI (TMS-EEG) analysis of network functional changes revealed a significant difference between HC and SCD group in the evaluated parameters of connectivity (Wave Form Adherence (WFA, Figures 2A,D; *p* < 0.05) and excitability (Area Under the Curve (AUC) of response, Figures 2B,D; *p* < 0.05). No significant difference was recorded between SCD and aMCI, however, a significant decrease was observed between SCD and Dementia subjects in the STP and WFA (Figures 2A,C,D; *p* < 0.05), as well as between HC and Dementia subjects (Figures 2A–D; *p* < 0.001) and HC and aMCI (Figures 2A–D; *p* < 0.01), in all DELPHI parameters, including STP (short term plasticity). DELPHI measures are in line with the changes observed in the WM integrity, both FA and MD, demonstrating high sensitivity of both DELPHI physiological measures of brain network integrity and WM changes to reflect early changes in SCD, prior to evident cognitive decline as observed in the aMCI and Dementia groups. This observation is consistent with previous data demonstrating WM changes in SCD prior to evident cognitive decline (10–14).

## Discussion

**Figure 3:**
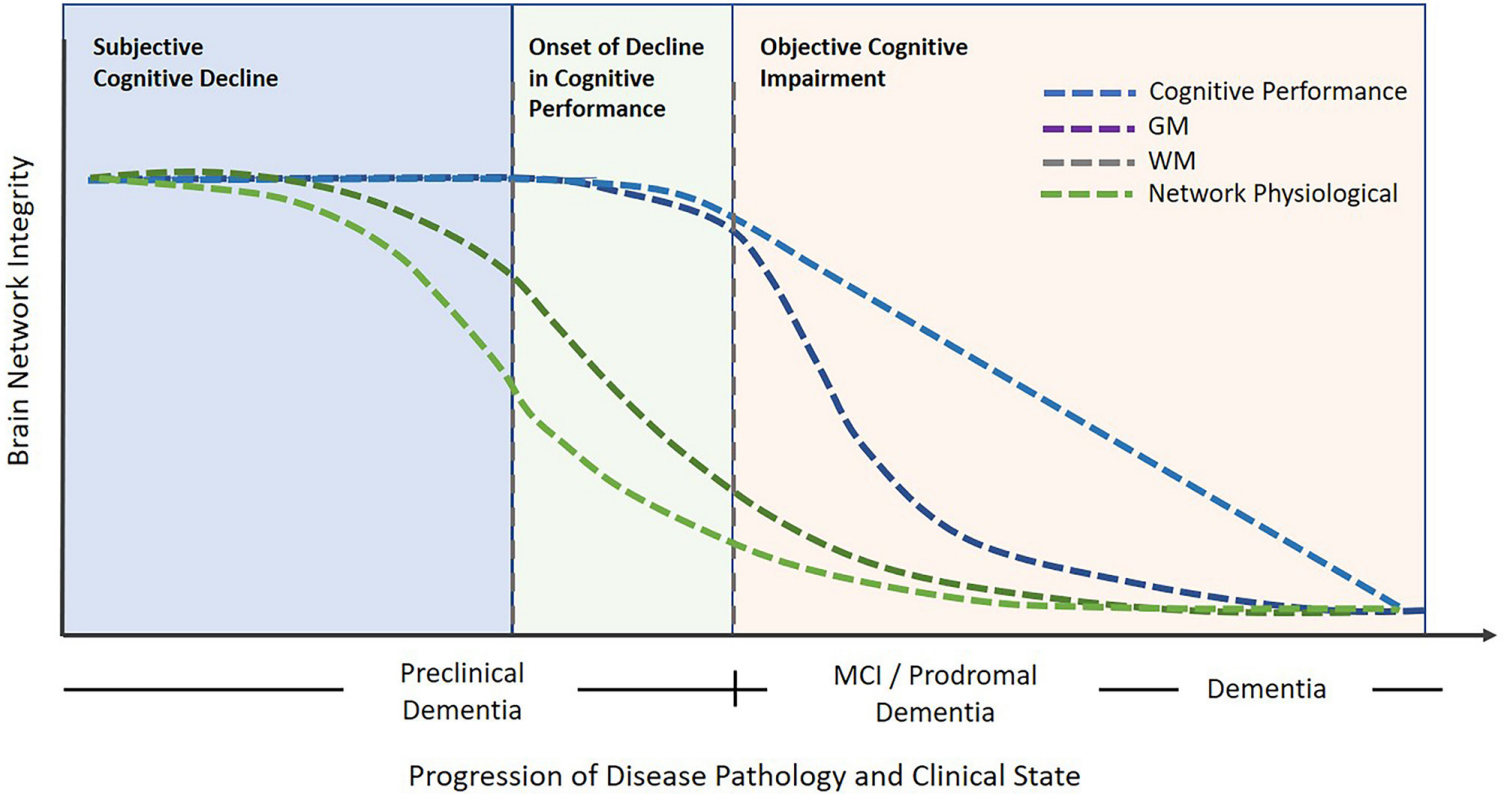
An illustrative model describing brain network integrity vs. cognitive decline in relation to progressive degeneration. Objective cognitive impairment is noticeable following the subjective cognitive impairment stage. In contrast, the early pathological phase of deterioration is characterized by neurophysiological and WM network changes. Subsequently, cognitive decline may progress onward to the stages of MCI and dementia which are evident with objective cognitive impairments and GM decrease. The blue dotted line represents cognitive performance changes, the purple dotted line represents Gray Matter changes, the olive dotted line represents White Matter changes, and the green dotted line represents Network physiological changes, The model principle was inspired and adapted from Jessen et al. (6).

The data presented in this study display brain network functional connectivity and plasticity as potential biomarkers for pre-clinical pathological degeneration. Study results indicate that although no pathological cognitive changes were detected in the SCD population compared to healthy norms, brain network functional and structural connectivity measured by DELPHI (TMS-EEG) and DTI in SCD subjects were significantly changed. In contrast, SCD subjects did not display any significant decrease in GM volume of specific ROIs related to cognitive neurodegeneration. A significant decrease in GM volume was evident only in aMCI and dementia groups compared to HC and SCD. This data supports the concept that changes in brain network function and microstructure connectivity precede manifestation in gray matter and objectively measured cognitive performance. Previous studies have also displayed such neurophysiological and microstructural disruptions prior to structural atrophy in individuals with SCD in parietal, frontal and hippocampal areas (31, 32). Interestingly, both WM and EEG-TMS measures of brain function display no significant differences between SCD and aMCI. These results may be caused by the limited number of subjects in the aMCI group or by the fact that the SCD group included subjects that were actively seeking treatment for their subjective cognitive decline and maybe at more progressive stages than other SCD subjects. In this study, no significant changes were found in WM, GM, and neurophysiological measures between aMCI and dementia groups. These results are supported by Kiuchi et al. (33, 34) but are not consistent with other studies that display WM changes between aMCI and dementia patients (35) and may be caused by the ROI-based analysis and small sample size of both the aMCI and dementia groups. These results may indicate that although the cognitive decline between aMCI and dementia is evident, the majority of neurophysiological and brain tissue damage has occurred before cognitive performance is compromised and may be objectively diagnosed. The results of this study, together with previous work, help support the understanding that brain network integrity deterioration precedes objective cognitive impairment and might be subjectively experienced by the patient. This concept is indactive of the early clinical manifestation of neurophysiological and microstructural changes as described schematically in Figure 3. This scheme [adapted and expanded from Jessen et al. 2014 (6)] displays objective cognitive performance deterioration during dementia progression compared to physiological clinical measures as presented in this study. Figure 3 illustrates the objective decrease in cognitive performance compared to GM volume decrease which is most prominent in objective cognitive impairment stages of aMCI and dementia vs. WM and neurophysiological measures which have been shown to display significant changes even in subjective cognitive decline subjects and progress in a slower rate in aMCI and Dementia groups (Figure 3). This figure illustrates the notion that by the time a disease or a cognitive impairment diagnosis is made most of the neuronal damage reflecting brain network integrity has already occurred. Therefore, the subjective perception of the patient might be sufficient to indicate the need for additional tests of these early neurophysiological biomarkers utilizing advanced technologies such as DELPHI (EEG-TMS) or DTI imaging and potentially prevent further degenerative deterioration.

It is important to note that, cognitive complaints in both SCD and aMCI may be caused by various factors such as depression, anxiety, and others, part of which may result in neurodegeneration and progress to different dementia types. These non-degenerative factors should be identified as they are treated and handled differently from neurodegenerative diseases. In addition, the current study compares different groups of subjects; healthy, SCD as well as aMCI and dementia representing subjects at different stages of cognitive impairment related to neurodegeneration. As this study is not a longitudinal study, in order to support its clinical findings and relevance, further longitudinal follow-up studies utilizing these clinical tools with controlled mood, cognitive, and other risk factors evaluations are required. In addition, the small sample size for each subject group in this study requires further validation in larger study groups. Nonetheless, the data presented in this study provides evidence supporting previous studies displaying noticeable physiological changes in brain network connectivity and function in earlier stages than detected nowadays using currently available standard of care tools as cognitive assessment and imaging tools (MR or CT) for structural brain imaging (36, 37).

## Conclusions

Detection of at-risk states and early disease stages are crucial for targeted and effective neurodegenerative dementia prevention. The data presented in this study suggest potential early detection biomarkers of changes in brain network integrity (connectivity and functionality) utilizing DTI or DELPHI (TMS-EEG) modalities. These data support our previous study that demonstrated the high correlation of DELPHI neurophysiological measures to WM integrity (38) and supports physiological evidence which singles out the SCD population as being at high risk for developing MCI and dementia. These tools and biomarkers may indicate early physiological changes in such at-risk and early disease stages, thus provide clinically available early-stage biomarkers of neurodegenerative related changes in subjects that have not yet displayed any other objective measurable cognitive deficits or GM volume decrease and facilitate the prevention of further neurodegenerative deterioration.

## Data Availability Statement

The original contributions presented in the study are included in the article/supplementary material, further inquiries can be directed to the corresponding author/s.

## Ethics Statement

The studies involving human participants were reviewed and approved by Shamir Medical center Ethical Committee review board. Written informed consent for participation was not required for this study in accordance with the national legislation and the institutional requirements.

## Author Contributions

HF, ID, and DT have determined the study design and criteria. NZ and TH conducted most of the laboratory works. OL-L and HF designed most of the DELPHI software algorithm. HF, DT, and ID contributed to designing and writing most of the manuscript and submitting the manuscript for publication. SE was the principal investigator in the study. GS, DH, and SE contributed to assuring the methods, the quality of the results, reviewing the manuscript, and approving for publication of the content. All authors contributed to the article and approved the submitted version.

## Conflict of Interest

HF, OL-L, NZ, TH, DH, DT, and ID have financial conflicts of interest with QuantalX neuroscience. The remaining authors declare that the research was conducted in the absence of any commercial or financial relationships that could be construed as a potential conflict of interest.

## Publisher's Note

All claims expressed in this article are solely those of the authors and do not necessarily represent those of their affiliated organizations, or those of the publisher, the editors and the reviewers. Any product that may be evaluated in this article, or claim that may be made by its manufacturer, is not guaranteed or endorsed by the publisher.
